# Synergistic Effects Between Mixed Plastics and Their Impact on Pyrolysis Behavior and Pyrolysis Products

**DOI:** 10.3390/molecules29246059

**Published:** 2024-12-23

**Authors:** Yong Li, Shengming Kang, Wenwen Han, Fengfu Yin

**Affiliations:** 1 College of Electromechanical Engineering, Qingdao University of Science and Technology, Qingdao 266061, China; ly15552797718@163.com (Y.L.); 15266932609@163.com (S.K.); 2 National Engineering Research Center of Advanced Tire Equipment and Key Materials, Qingdao University of Science and Technology, Qingdao 266061, China

**Keywords:** mixed plastics, synergistic effect, kinetic analysis, products analysis, pyrolysis mechanism

## Abstract

Pyrolysis is recognized as a promising technology for waste plastics management. Although there have been many studies on pyrolysis of waste plastics, there is still a lack of in-depth research on the mechanism of synergistic effect between mixed plastics and the mechanism of product formation. In this paper, based on the pyrolysis characteristics of Polystyrene, Polyethylene, and mixed plastics (Polystyrene/Polyethylene), it is demonstrated that a synergistic effect exists in the co-pyrolysis of Polystyrene/Polyethylene and affects the pyrolysis behavior and pyrolysis products. It was found that polystyrene chain segments containing C=C double bonds, generated from the pyrolysis of polystyrene, initiated the pyrolysis of polyethylene during the polystyrene/polyethylene co-pyrolysis, resulting in the termination pyrolysis temperature of the co-pyrolysis being advanced by 19.8 K. Due to the reduction in the termination pyrolysis temperature by 19.8 K, the average activation energy of the co-pyrolysis was reduced by about 14%. Compared with the weighted values of single-component plastics (Polystyrene and Polyethylene), the actual oil production of co-pyrolysis increased by 9.7% to 89.80%. At the same time, the content of low molecular weight Styrene and Toluene in pyrolysis oil increased by 12.3% and 1.65%, respectively. This study provides a useful and comprehensive reference for realizing the closed cycle of “from plastics to plastics”.

## 1. Introduction

Global plastic production has increased significantly since 1950, from 3 million tons in 1953 to 391 million tons in 2021. It is predicted that if this trend is not changed, the annual global production of plastic could increase to 1.8 billion tons by 2050 [1,2]. In daily life, Polyethylene (PE), Polypropylene (PP), Polyvinyl chloride (PVC) and Polystyrene (PS) are the most common types of waste plastics, which together account for about 70% of the total waste plastics [3,4]. However, the main treatment methods are still landfill (79%) and incineration (12%). Such treatment will not only occupy a large amount of land resources, but also cause irreversible damage to the ecological environment and human health due to the pollutants released by incineration [5,6]. Therefore, there is an urgent need for an environmentally friendly technology to facilitate the high-value recycling of waste plastics.

Physical recycling and pyrolysis technology are the two main ways to reuse waste plastics [7,8,9]. Although the physical recycling of waste plastics is widely used due to its ease of operation, the method has significant limitations. In particular, after repeated physical recycling, the performance of plastics will continue to decline, so the way of physical recycling is not the ultimate solution for waste plastic disposal. Due to the high volatile content and low ash content of plastics, pyrolysis technology can effectively transform waste plastics into liquid fuels, chemical raw materials, or new plastic products. Therefore, pyrolysis technology is regarded as one of the most promising methods for the treatment of waste plastics [10,11,12,13,14]. However, in the pyrolysis process of waste plastics, factors such as the type of plastic, mixing ratio, pyrolysis temperature, holding time, and heating rate are all crucial factors influencing industrial production of waste plastics [15,16,17,18]. Additionally, the pyrolysis technology of waste plastics is divided into conventional pyrolysis, catalytic pyrolysis, and other methods [19,20]. Therefore, considering the economic feasibility and applicability of industrial pyrolysis of waste plastics, it is urgent to select suitable types of waste plastics, reduce the pyrolysis temperature, increase the yield of pyrolysis oil, and obtain high-value-added products. Numerous studies have found that different types of waste plastics face varying degrees of challenges during pyrolysis. During the pyrolysis of PVC, the hydrochloric acid produced not only corrodes the pyrolysis equipment, but also poses a new threat to the environment if not handled properly [21]. When pyrolyzing PP, the composition of the pyrolysis oil is more complex compared to PE, which increases the difficulty of recycling the pyrolysis oil [22]. According to reports, the co-pyrolysis of waste plastics PS and PE lowers the termination pyrolysis temperature during the process [23,24,25,26]. Although the co-pyrolysis of PS and PE has a positive impact on pyrolysis behavior, existing studies still lack sufficient information to clarify the interactions between PS and PE during co-pyrolysis. Therefore, it is necessary to conduct more in-depth research on the synergistic effects and product formation mechanisms during the co-pyrolysis of PS and PE to better understand the specific impact of co-pyrolysis on pyrolysis behavior and product formation.

In this study, we investigated the pyrolysis characteristics of single component plastic PS and PE, and mixed plastic PS/PE. It was confirmed that there is a positive synergistic effect of mixed pyrolysis, and this synergistic effect contributes to the increase in pyrolysis oil yield. On this basis, we performed gas chromatography–mass spectrometry (GC-MS) tests on the pyrolysis oils, and Fourier transform infrared spectrometry (FTIR) tests of the experimental materials (PS, PE, mixed plastics PS/PE), and the products of the pyrolysis of mixed plastic PS/PE at 697.15 K. It was found that in the pyrolysis system of mixed plastic PS/PE, the presence of PS initiated the early pyrolysis of PE and reduced the termination pyrolysis temperature and the activation energy required for pyrolysis of mixed plastic PS/PE. The presence of PE delayed the pyrolysis of PS and increased the yield of lower molecular weight Styrene and Toluene in the pyrolysis oil. This study provides a useful and comprehensive reference for achieving high-value treatment of waste plastics.

## 2. Results and Discussion

### 2.1. Characteristics of Waste Plastics

Table 1 demonstrates the proximate and elemental analysis of waste plastics. As shown in Table 1, PS and PE contain abundant carbon and hydrogen, and the content of volatiles is extremely high, up to more than 99.92%. Thus, a liquid or gaseous product is easily obtained from pyrolysis [27]. 

### 2.2. Pyrolysis Characteristics Analysis

**Figure 1:**
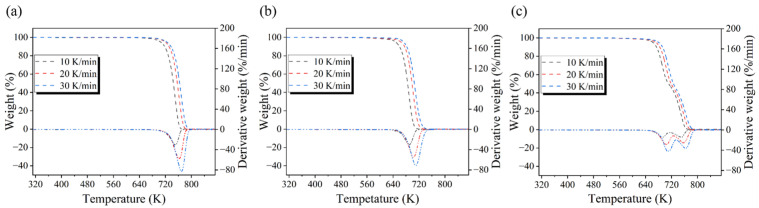
(**a**) TG and DTG curves for the pyrolysis of PE, (**b**) TG and DTG curves for the pyrolysis of PS, (**c**) TG and DTG curves for the pyrolysis of PS/PE.

According to the test results of the TGA, the TG and DTG curves of the experimental samples were obtained. The TG and DTG curves of the pyrolysis of single-component plastics (PE, PS) and mixed plastics (PS/PE) at different heating rates are shown in Figure 1. Pyrolysis characteristics are a further explanation of the TG and DTG curves [28]. According to the pyrolysis characteristics of the experimental samples, the experimental conditions of the pyrolysis reactor can be set more reasonably. The pyrolysis characteristics of PE, PS, and PS/PE at different heating rates are shown in Table 2.

From the TG and DTG curves in Figure 1, it can be seen that the pyrolysis of the single component (PE, PS) was accomplished as a one-step reaction. Due to their similar molecular structure, PE and PS exhibited the same pyrolysis tendency. However, in the pyrolysis process of mixed plastics (PS/PE), two weight loss peaks appeared; the fastest pyrolysis rate corresponding to the first weight loss peak occurs at a temperature of 691.75 K and is caused by PS pyrolysis. The fastest weight loss rate of the second weight loss peak corresponds to a pyrolysis temperature of 730.5 K, which is mainly caused by PE pyrolysis.

The pyrolysis characteristics of the experimental samples at 10, 20, and 30 K/min were analyzed based on the data shown in Figure 1 and Table 2. At a heating rate of 10 K/min, the pyrolysis temperature ranges of one-component PE and PS were 728.05~757.45 K and 667.65~702.25 K, respectively. At a heating rate of 20 K/min, the pyrolysis temperature ranges of one-component PE and PS were 744.05~761.35 K and 680.65~716.75 K, respectively. At a heating rate of 30 K/min, the pyrolysis temperature ranges of one-component PE and PS were 748.85~765.15 K and 687.75~724.05 K, respectively. It can be seen that with the increases in heating rate, the pyrolysis process moves to higher temperatures. The main factor causing this phenomenon was the hysteresis of heat transfer that occurs during thermal decomposition [29]. Compared to PE, the pyrolysis of PS can be completed at relatively low temperatures, which indicates that PE has better thermal stability than PS. The reason is that the molecular structures of PE and PS are different; PE has a straight chain molecular structure, while PS has a larger side chain with a benzene ring. The pyrolysis temperature ranges of mixed plastics (PS/PE) under three different heating rates are 668.75~737.65 K, 678.05~751.75 K, and 696.55~757.45 K, respectively. Moreover, the final pyrolysis temperatures at each heating rate are lower than that of pure PE. This suggests that during the mixed pyrolysis process, PS accelerates the pyrolysis of PE, allowing PE to complete the pyrolysis reaction at a lower temperature [30].

### 2.3. Synergy Analysis of Mixed Plastics

**Figure 2:**
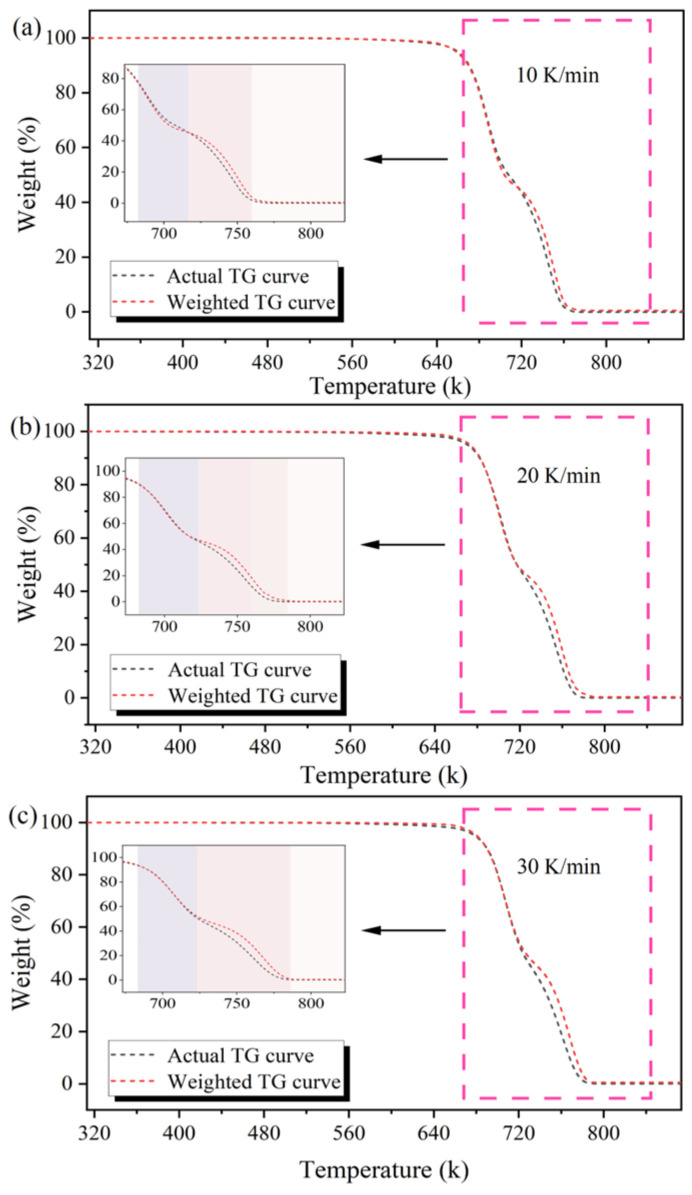
(**a**) The Actual and weighted TG curves at a heating rate of 10 K/min, (**b**) The Actual and weighted TG curves at a heating rate of 20 K/min, (**c**) The Actual and weighted TG curves at a heating rate of 30 K/min.

PS and PE were mixed for pyrolysis, and the influence of the synergistic effect between them on the pyrolysis characteristics, pyrolysis kinetics, and pyrolysis products of the whole sample was investigated [31]. The type of synergistic effect and the synergistic temperature range in the PS/PE mixed pyrolysis process were analyzed by using the degree of overlap between the actual TGA curves and the weighted TGA curves obtained from Equation (1). The synergistic effects at three different heating rates (10, 20, and 30 K/min) are shown in the Figure 2. The results show that the synergistic effect is significantly influenced by the heating rate. Specifically, an increase in heating rate makes the synergistic effect more pronounced. However, changes in the heating rate do not alter the type of the synergistic effect. They only affect the temperature range in which the synergistic effect occurs, shifting it to higher temperature ranges. Therefore, this study analyzes the synergistic effect at a heating rate of 10 K/min.

Figure 2a shows that when the pyrolysis temperature ranges from 668.75 to 713.15 K, the actual conversion rate of PS/PE mixed pyrolysis is slightly lower than the weighted conversion rate (ΔWTG < 0), indicating that the synergistic effect of PS/PE mixed pyrolysis in this temperature range is negative. However, based on the pyrolysis characteristics of PE, it can be observed that the temperature range does not reach the initial pyrolysis temperature of PE. Therefore, the negative synergistic effect in this temperature range is caused by the mutual covering of PS and PE during mixing, which delays the pyrolysis of PS. As the temperature continues to rise, the pyrolysis oil covering the surface of the mixed plastic quickly vaporizes into pyrolysis gas and separates. The actual conversion rate begins to exceed the weighted conversion rate (ΔWTG > 0). This indicates that a positive synergistic effect begins to emerge in the PS/PE mixed pyrolysis process. It can also be observed that the actual TG curve completes the pyrolysis earlier than the weighted TG curve. This suggests that a certain type of substance generated during PS pyrolysis triggers the early pyrolysis of PE. In addition, the actual residue mass (0.20%) produced can be found through Figure 2 to be less than the weighted residue mass (0.22%), indicating that the overall synergistic effect of PS/PE is positive synergistic.

### 2.4. Kinetic Analysis

**Figure 3:**
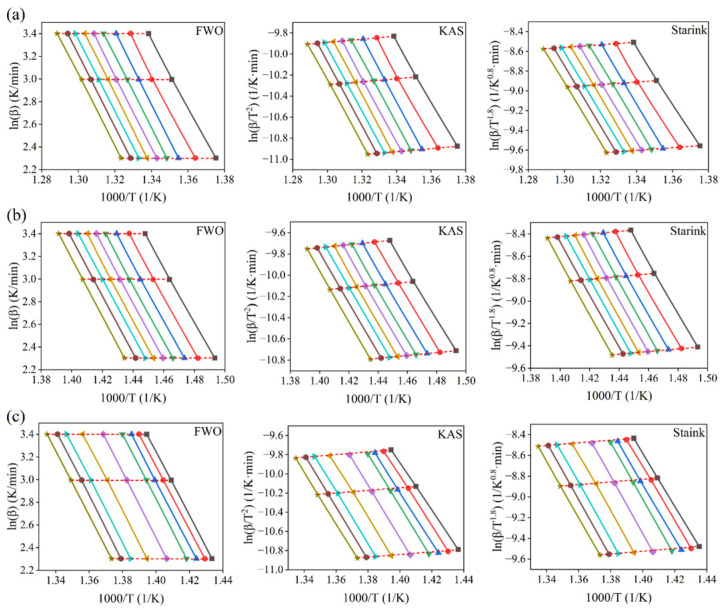
(**a**) The fitting curves for three model-free methods for PE, (**b**) The fitting curves for three model-free methods for PS, (**c**) The fitting curves for three model-free methods for PS/PE. (The colorful lines in the graph from left to right indicate a conversion rate of 0.9~0.1).

The minimum energy required to initiate a chemical reaction or produce an activated complex is called activation energy. A lower activation energy indicates that the experimental sample only needs less energy from the outside world to achieve a higher pyrolysis reaction during the pyrolysis process [32]. The conversion rate (α) selected in this experiment ranged from 0.1 to 0.9, and the interval was 0.1. The TG data of single component plastic PS, PE, and mixed plastic PS/PE obtained at three heating rates of 10 K/min, 20 K/min, and 30 K/min were substituted respectively into the FWO equation, KAS equation, and Starink equation, and then curve fitting was carried out with 1000/T as the horizontal axis and ln(β), ln(β/T^2^), and ln(β/T^1.8^) as the vertical axis. The fitting curves of these three model-free methods for each experimental sample of PE, PS, and PS/PE are shown in Figure 3. It can be seen from Figure 3 that in the curve fitting process, the slopes of the fitting curves of the different kinetic models were basically consistent, indicating that only one pyrolysis mechanism function model was followed in the pyrolysis process of PE, PS, and PS/PE. This indicates that the pyrolysis reaction of the three samples tends toward a dominant mechanism, with the reaction process being relatively simple and uniform. It also indicates that their structures have certain similarities.

Based on the slope of the fitted curve in Figure 3, the activation energy (E_α_), correlation coefficient (R_2_), and average activation energy (E_α_) of PE, PS, and PS/PE at different conversion rates can be obtained by using Equations (7)–(9). The calculation results are shown in Table 3, Table 4 and Table 5.

As can be seen from Table 3, Table 4 and Table 5, the correlation coefficients (R_2_) in the pyrolysis process of PE, PS, and PS/PE were all greater than 0.999, which indicated that the activation energy (E_α_) estimated by the three model-free methods had high accuracy [33]. In addition, there was little difference between the E_α_ and R_2_ estimated by the three model-free methods, which indicated that the results of the three model-free methods were reliable. This study first employed three different model-free methods to determine the kinetic parameters of waste plastics, with the aim of reducing the calculation errors that may arise from using a single model-free method. Subsequently, by comparing the results of these three methods, it was found that the differences in estimating the activation energy (E_α_) and correlation coefficient (R_2_) were very small. This result also indicates that the three model-free methods are highly reliable. Therefore, to make the analysis process simpler and clearer, this study selected the FWO method from the three model-free methods to analyze the estimated results. It can be found that the E_α_ of each reaction under different conversion rates was different, which indicated that the E_α_ of the experimental sample pyrolysis varied with the change in conversion rate α [34]. By the model-free method, the E_α_ of PE, PS, and PS/PE changed in the range of 234.7~255.8 kJ, 189.4~200.5 kJ, and 191.1~229.5 kJ, respectively. These results are slightly different from the activation energy required for PE pyrolysis reported by Aboulkas et al. [35], which is 238~247 kJ, and the activation energy required for PS pyrolysis reported by Ozsin et al. [36], which is 203.1~227.4 kJ. These slight differences can be attributed to factors such as differences in sample size, changes in gas flow rates during TG testing, and so on. The difference in Eα between PE and PS is due to the difference in molecular structure. PS is polymerized from styrene monomer, and its molecular structure contains benzene rings. During the pyrolysis of PS, the conjugated structure of the benzene ring can, to some extent, promote the cleavage of PS molecular chains by releasing free radicals, which helps reduce the activation energy required for pyrolysis. In contrast, PE is formed by the polymerization of ethylene monomers, and its molecular structure is primarily composed of saturated hydrocarbon chains (-CH_2_-CH_2_-). The cleavage of molecular chains in this structure requires more energy, resulting in a relatively higher activation energy for PE. In addition, the average activation energy during pyrolysis of the mixed plastic PS/PE is reduced by about 14% compared to the average activation energy of the single-component plastic PE.

### 2.5. Distribution and Proportion of Pyrolysis Products

The pyrolysis experiments were carried out in the pyrolysis reactor, and the yield and proportion of pyrolysis products of different experimental samples were obtained. As can be seen from Table 6, the yield of pyrolysis oil was in the order of PS/PE > PS > PE, and the yield of pyrolysis gas was PE > PS > PS/PE, while there was a slight difference in the residue. Obviously, PS/PE mixed plastic pyrolysis produced more pyrolysis oil and less pyrolysis gas than PE and PS. This is because the co-pyrolysis of mixed plastics PS/PE results in a 19.8 K decrease in the termination temperature, which means that mixed plastics PS/PE can undergo pyrolysis at a lower temperature. Lower pyrolysis temperatures tend to favor the production of liquid products while reducing the likelihood of gaseous products [37]. Compared to the weighted data for single component plastics (PS, PE), the actual oil yield from hybrid pyrolysis increased by 9.7%.

### 2.6. GC-MS Analysis of Pyrolysis Oil and Formation Mechanism

**Figure 4:**
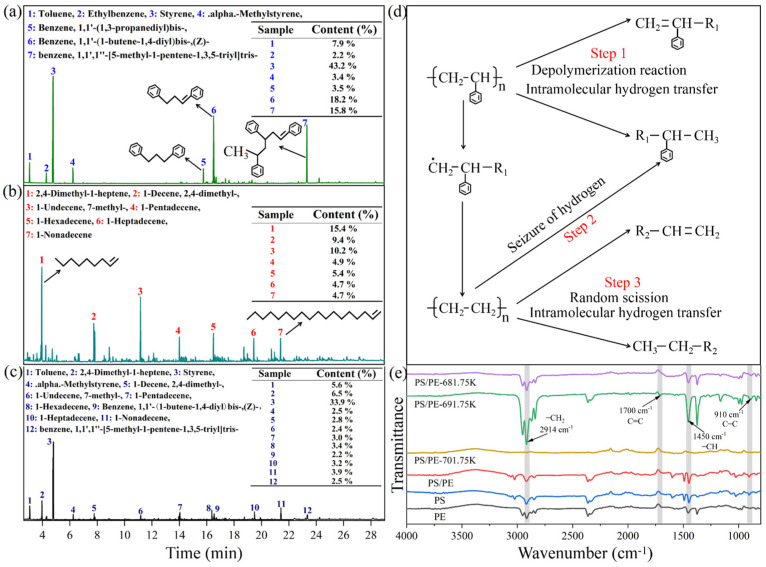
Panels (**a**–**c**) show gas chromatography–mass spectrometry of the pyrolytic oils of the single-component plastic PS and PE, and the mixed plastic PS/PE, respectively. (**d**) The pyrolysis mechanism diagram of mixed plastic PS/PE is shown. (**e**) Fourier transform infrared spectra are shown, where PE, PS, and PS/PE represent the FTIR spectra of raw materials, respectively, PS/PE-681.75 K represents the FTIR spectra of pyrolysis products after pyrolysis of mixed plastics at 681.75 K; PS/PE-691.75 K represents the FTIR spectra of pyrolysis products after pyrolysis of mixed plastics at 691.75 K; and PS/PE-701.75 K represents the FTIR spectra of pyrolysis products after pyrolysis of mixed plastics at 701.75 K.

The pyrolysis temperature has a great influence on the pyrolysis products. As the pyrolysis temperature increases, the activity of intermediates generated in the initial pyrolysis reaction increases, and the complexity of the whole pyrolysis reaction increases, such as secondary reaction, dehydrogenation, cyclization, etc. [38]. In this study, the main components of the pyrolysis oil of single-component plastics (PS, PE) and mixed plastics (PS/PE) with the content of more than 2% were selected for comprehensive analysis. The content of each component was obtained by normalization of peak area. Figure 4a–c show gas chromatography–mass spectrometry of the pyrolytic oils of single-component plastics PS, PE, and mixed plastics PS/PE, respectively. As shown in Figure 4a, the main products in the pyrolysis oil of PS were aromatic compounds (C_7_~C_24_). Among them, the content of Styrene (43.2%) and Benzene, 1,1′-(1-butene-1,4-diyl)bis-, (Z)- (18.2%) was the highest. Similar reports have also been made by other researchers [39,40]. As shown in Figure 4b, the main product in the pyrolysis oil of PE was olefin (C_9_~C_18_). Among them, the content of 2,4-Dimethyl-1-heptene (15.4%) and 1-Undecene, 7-methyl-(10.2%) were the highest. The study by Zhang et al. [38] also reported similar results. The formation of these products in PS and PE pyrolysis oils is attributed to the random cleavage of molecular chains during pyrolysis, as well as inter- or intramolecular hydrogen transfer reactions. After PE and PS were mixed 1:1 according to mass, the highest content in the pyrolysis oil was Styrene (33.9%), as shown in Figure 4c. To further quantify the effect of mixed pyrolysis on the products. The yield of pyrolysis products of single-component plastics was weighted proportionally, and the weighted value was compared with that of the actual pyrolysis products of mixed plastics. It was found that the yield of Styrene (33.9%) in the pyrolysis oil of mixed plastics increased by 12.3% compared with the weighted value (21.6%). The yield of Toluene (5.6%) compared with the weighted value (3.95%) increased by 1.65%; the yield of Benzene,1,1′-(1-butene-1,4-diyl)bis-, (Z)- (2.2%) compared with the weighted value (9.1%) increased by 6.9%; and the yield of Benzene,1,1′,1″-[5-methyl-1-pentene-1,3,5-triyl]tris- (2.5%) compared with the weighted value (7.9%) decreased by 5.4%. This is because in the pyrolysis process of mixed plastics, the presence of PE increases the pyrolysis temperature of PS in the whole pyrolysis system. The large molecular weight PS chain segment (e.g., Benzene, 1,1′-(1-butene-1,4-diyl)bis-, (Z)-, benzene and 1,1′,1″-[5-methyl-1-pentene-1,3,5-triyl]tris-, etc.) is further broken to form low molecular weight Toluene, Styrene, etc. It was also observed in the pyrolysis oil that the olefin yield of C_9_~C_14_ was lower than the weighted value, while that of C_15_~C_18_ was higher than the weighted value. This is because in the pyrolysis process of mixed plastics, the PS pyrolysis reaction occurred first and then the PS chain segment with a certain molecular weight was produced. The PS chain segment with a certain molecular weight seized hydrogen on the PE, and initiated and accelerated the depolymerization of PE [25]. Therefore, mixed pyrolysis of PS and PE helps to reduce the pyrolysis temperature, and then reduced the depolymerization reaction of the macromolecular PE chain segment (C_15_~C_18_).

To investigate the mechanism of synergistic effect between the blended plastics, we carried out FTIR spectroscopy of the pyrolysis products of PS/PE blended plastics at 681.75 K, 691.75 K, and 701.75 K. Additionally, for comparative analysis, FTIR spectroscopy was also performed on the experimental materials PS, PE, and PS/PE. As shown in Figure 4e, no distinct C=C double bond characteristic peak was detected in the pyrolysis products at 681.75 K. This may be because the C=C double bond content generated by the PS/PE mixed plastics at this temperature is low and could not be detected. Similarly, no distinct C=C double bond characteristics were observed at 701.75 K. It is speculated that at this temperature, PS has almost completed pyrolysis, and the PS chain segments containing C=C double bonds are expelled from the quartz boat and exist in the condenser as pyrolysis oil, thus not being detected. In addition, the results show that the stretching vibration peaks of -CH_2_, -CH, and C-C are observed at 2914 cm^−1^, 1450 cm^−1^, and 910 cm^−1^, respectively. These characteristic peaks are present in the experimental materials PS, PE, mixed plastic PS/PE, and the pyrolysis products of mixed plastic PS/PE. However, after the pyrolysis of mixed plastic PS/PE at 691.75 K, a unique C=C double bond stretching vibration peak appeared at 1700 cm^−1^ in its products. This indicates that the pyrolysis of PS generates PS chain segments containing C=C double bonds. These segments can capture hydrogen (H) from PE, thereby initiating the pyrolysis reaction of PE. The pyrolysis mechanism of mixed plastic PS/PE is shown in Figure 4d. During the pyrolysis of mixed plastic PS/PE, due to the low pyrolysis temperature of PS, depolymerization and hydrogen transfer reactions of PS first occurred (Step 1). Then, PS segments with a certain molecular weight (such as Toluene; Ethylbenzene; Benzene, 1,1′-(1,3-propanediyl)bis-, etc.); and PS segments with C=C double bonds with a certain molecular weight (such as Styrene and Benzene, 1,1′-(1-butene-1,4-diyl)bis-, (Z)-,) were generated. As the temperature increased, PS chain segments containing active radicals and having a certain molecular weight robbed the hydrogen on PE (Step 2). It intensified the depolymerization reaction and hydrogen transfer reaction of PE (Step 3), and olefin (C_9_~C_18_) with a certain molecular weight was generated. Therefore, the presence of PS reduced the pyrolysis temperature of PE, thus reducing the activation energy of the whole pyrolysis system of mixed plastics. However, the presence of PE indirectly increased the pyrolysis temperature of PS in the whole pyrolysis system, making the PS chain segment with large molecular weight further break to form small molecular weight aromatic hydrocarbons.

## 3. Materials and Methods

### 3.1. Experimental Samples

In the study of pyrolysis of waste plastics, using pure plastics instead of waste plastics can enhance the reference value of experimental results. The plastic samples in this experiment were procured from the Shandong branch of China Petrochemical Corporation. PS and PE were pulverized using a crusher (FW117, Taisite Instruments Company, Tianjin, China). Plastic particles with a particle size of less than 200 mesh were screened, and then PS and PE were mixed according to a mass ratio of 1:1, thus obtaining the mixed plastic PS/PE. The mixed plastic samples were used for TGA tests and pyrolysis reactor experiments. According to the GB/T212-2008 standard, a muffle furnace (FO410C, Daiwa, Shanghai, China) was deployed for proximate analysis of plastic samples to determine moisture (M), volatile matter (VM), fixed carbon (FC), and ash content [41]. An elemental analyzer (Vario EL III, Element Trading Company, Shanghai, China) was used to analyze plastic samples for elements, including carbon (C) and hydrogen (H).

### 3.2. Thermogravimetric Analysis (TGA)

TGA (209 F3, Netzsch Company, Bavaria, Germany) was adopted to investigate the pyrolysis characteristics of plastics at 313.15~1073.15 K, in which the heating rates were 10, 20, and 30 K/min and the mass of the individual test samples was 10~15 mg. To prevent sample oxidation, purge gas at a flow rate of 50 mL/min and protective gas at a flow rate of 20 mL/min were used to remove other impurities from the sample chamber before the formal test.

### 3.3. Synergistic Effects of Mixed Plastics Pyrolysis

The overlap of the weighted TGA curve and actual TGA curve was used to determine the type of synergistic effect and synergistic temperature range in the mixed plastic pyrolysis process. The type of synergistic effect can help to identify plastic combinations that reduce energy consumption [42].

The weighting formula is as follows [43]:

(1) $${TG}_{C}=\sum_{i=1}^{n}\mu_{i}\cdot W_{i}$$

(2) $$∆W_{TG}={TG}_{E}-{TG}_{c}$$

where µ_i_ is the proportion of single-component plastics to the total mass of the mixed plastics. W_i_ is the TG curve of the single-component plastics. TG_E_ and TG_C_ are the actual and the weighted TG curves of mixed plastics, respectively. ΔW_TG_ is the difference between the actual and weighted curves. At a certain pyrolysis temperature, if the weighted TG curve is higher than the experimental curve (i.e., the actual conversion rate is higher than the predicted value), it indicates that there is a positive synergistic effect between the mixed plastics at that temperature. On the contrary, if the weighted TG curve at a certain pyrolysis temperature is lower than the experimental curve (i.e., the actual conversion rate is lower than the predicted value), it indicates that there is a negative synergistic effect between the mixed plastics at that temperature.

### 3.4. Calculation Formula of Kinetic Parameters

The calculation formula for the conversion rate α during the pyrolysis process of the plastic sample is as follows:

(3) $$\alpha =\frac{m_{0}-m_{t}}{m_{0}-m_{\infty }}$$

where α is the conversion rate (%), m_0_ is the initial mass (mg), m_t_ is the sample mass at temperature t (mg), and m_∞_ is the final residue mass (mg).

The reaction rate (dα/dt) is governed by the reaction rate constant k(T) and the pyrolysis mechanism function f(α). The relationship between k(T) and temperature T is expressed by the Arrhenius equation:

(4) $$\frac{d\alpha }{dt}=k(T)f(\alpha )=Aexp(-\frac{E_{\alpha }}{RT})f(\alpha )$$

where t stands for time (min), f(α) is the differential expression of the pyrolysis mechanism function, A is the pre-exponential factor (min^−1^), E_α_ denotes the activation energy (kJ), and R is the universal gas constant (8.314 J).

The relationship between T and t in a non-isothermal process is as follows:

(5) β=dT/dt

where β is the heating rate (K/min). Substituting Equation (5) into Equation (4), the comprehensive expression for the non-isothermal, non-homogeneous reaction of the test sample is obtained:

(6) $$\frac{d\alpha }{dT}=\frac{A}{\beta }exp(-\frac{E_{\alpha }}{RT})f(\alpha )$$

The model-free methods are considered an effective tool for estimating kinetic parameters. This method does not require assuming a pyrolysis mechanism function model to obtain the kinetic parameters of the experimental samples. This study employed three model-free methods to determine the kinetic parameters. This approach avoids the relative errors in the calculation process of a single model-free method, ensuring the reliability of the results [43].

The equation for the Flynn–Wall–Ozawa (FWO) method is expressed as follows [42]:

(7) $$\ln\left(\beta \right)=-\frac{1.052E_{\alpha }}{RT}+\ln\left(\frac{AE_{\alpha }}{RG\left(\alpha \right)}\right)$$

where G(α) is the integral expression of the pyrolysis mechanism function.

The equation for the Kissinger–Akahira–Sunose (KAS) method is expressed as follows [44]:

(8) $$\ln\left(\frac{\beta }{T^{2}}\right)=-\frac{E_{\alpha }}{RT}+\ln\left(\frac{AR}{E_{\alpha }G\left(\alpha \right)}\right)$$

The equation for the Starink method is expressed as follows [45]:

(9) $$\ln\left(\frac{\beta }{T^{1.8}}\right)=-\frac{1.003E_{\alpha }}{RT}+\ln\left(\frac{AR}{E_{\alpha }G\left(\alpha \right)}\right)$$

The process of fitting curves in Equations (7)–(9) takes 1/T as the abscissa and ln(β), ln(β/T^2^), and ln(β/T^1.8^) as the ordinate, respectively. According to the slope of the fitting curve, the activation energy (E_α_) and correlation coefficient (R_2_) at different conversion rates are obtained.

### 3.5. Pyrolysis Experiments

**Figure 5:**
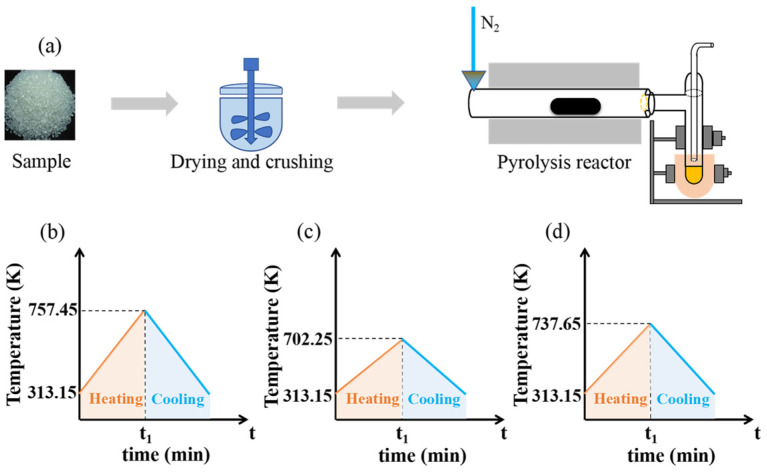
(**a**) Flow chart of pyrolysis experiments of PE, PS, and PS/PE; (**b**) temperature-rise curve set for PE pyrolysis; (**c**) temperature-rise curve set for PS pyrolysis; (**d**) temperature rise curve set for PS/PE pyrolysis.

To investigate the yield and distribution of PE, PS, and PS/PE pyrolysis products, pyrolysis experiments were carried out on the samples using a pyrolysis reactor (SK-G08123K-610, Tianjin Zhong huan Electric Furnace Co., Ltd., Tianjin, China). The pyrolysis reactor consists of a furnace body, quartz tube, quartz boat, vacuum sealing components, and an electric control box. The reactor has a rated power of 3 kW, a rated voltage of 220 V, and an adjustable heating rate of 0–10 K/min. The flow charts of PE, PS, and PS/PE pyrolysis are shown in Figure 5a. The pyrolysis experiments were carried out under a nitrogen atmosphere. Before the experiment began, the initial mass of the experimental sample, quartz boat, quartz tube, and condensing tube was weighed and recorded, and then the heating procedure of the pyrolysis reactor was set. The temperature was raised at a heating rate of 10 K/min to the termination pyrolysis temperature of PE. The pyrolysis experiment of single-component PE was labeled as experiment A. After the end of experiment A, the pyrolysis reactor was cooled to room temperature and the final mass of the quartz boat, quartz tube, and condensing tube was weighed. The pyrolysis oil in the condensate tube and quartz tube and the residue in the quartz boat were collected, and the mass and percentage of the pyrolysis oil, pyrolysis gas, and residue were calculated. According to the operation steps in experiment A, the pyrolysis experiment was carried out at a heating rate of 10 K/min to the termination pyrolysis temperature of PS. The pyrolysis experiment of a single component of PS was labeled as experiment B. Then, according to the operation steps in experiment A, the pyrolysis experiment was carried out by heating at a rate of 10 K/min to the terminal pyrolysis temperature of mixed plastic PS/PE. The pyrolysis experiment of mixed plastic PS/PE was labeled as experiment C. The mass of pyrolysis gas was calculated by subtracting the difference in pyrolysis oil and residue mass from the experimental sample.

### 3.6. GC-MS Tests of Pyrolysis Oil

A gas chromatography mass spectrometer (7890B-7000C, Agilent Technologies Company, Santa Clara, CA, USA) was utilized for compositional analysis of pyrolysis oil samples. The sample was first diluted with ethyl acetate (1:10) before analysis. Sampling was performed using a pipette, with a ratio of 1 part pyrolysis oil to 10 parts ethyl acetate, for a total sample volume of 1 mL, and the products were subsequently analyzed on a DB-5 column (30 m × 0.25 mm × 0.25 µm). Helium was used as the carrier gas at a flow rate of 1.0 mL/min. The oven temperature was initially set at 333.15 K and held for 2 min, then increased to 553.15 K at a heating rate of 10 K/min and held for 5 min with an injection volume of 1.0 µm. The chromatographic peaks were matched for identification using the NIST14 mass spectral library.

### 3.7. Fourier Transform Infrared Tests

This study aimed to investigate the pyrolysis products of PS in the mixed PS/PE system and their initiation effect on the pyrolysis of PE. For this purpose, we selected the pyrolysis products of PS/PE at three different temperatures (681.75 K, 691.75 K, and 701.75 K) and conducted Fourier transform infrared spectroscopy tests (FTIR, IS50, Thermo Technologies Company, Waltham, MA, USA). Additionally, for comparative analysis, FTIR spectroscopy tests were also performed on the experimental materials PS, PE, and PS/PE using scan times of 16 per minute and wavelength range of 400–4000 nm.

## 4. Conclusions

This study investigated the pyrolysis characteristics of single-component plastics (PS, PE) and mixed plastics (PS/PE), and the synergistic effects of mixed plastics during the pyrolysis process. The results show that the co-pyrolysis of mixed plastics not only affects the pyrolysis behavior but also influences the activation energy required for pyrolysis, the distribution of pyrolysis products, and the composition of the pyrolysis oil. Compared to single-component plastics (PE), co-pyrolysis lowers the termination pyrolysis temperature for mixed plastics (PS/PE) by 19.8 K and reduces the average activation energy by approximately 14%. Due to the reduction in termination pyrolysis temperature, the oil yield from co-pyrolysis of mixed plastics (PS/PE) increased by 9.7%, reaching 89.80%, compared to the weighted values of single-component plastics (PS, PE). In the pyrolysis system of mixed plastics (PS/PE), the presence of PE indirectly increased the pyrolysis temperature of PS. As a result, the content of low molecular weight styrene and toluene in the pyrolysis oil increased by 12.3% and 1.65%, respectively. The findings of this study contribute to enhancing the recycling and reutilization of waste plastics, providing strong technical support for the sustainable development of waste plastic management.

In future research, based on the findings and experimental data of this study, we will conduct more extensive experiments to determine the optimal operating conditions for industrial pyrolysis, aiming to promote the industrial application of mixedwaste PS/PE plastic pyrolysis technology. We also hope to provide practical and effective solutions for the management and resource utilization of waste plastics.

## Figures and Tables

**Table 1:** Physicochemical properties of PE, PS, and PS/PE.

Samples	Industrial Analysis (%)				Ultimate Analysis (%)				
	Ash	VM	M	FC	C	H	O	N	S
PS	0.04	99.92	0.00	0.04	92.44	7.20	0.36	-	-
PE	0.01	99.92	0.03	0.04	85.79	13.82	0.39	-	-
PS/PE	0.03	99.92	0.02	0.03	89.12	10.50	0.38	-	-

**Table 2:** The pyrolysis characteristics of PE, PS, and PS/PE at different heating rates.

Samples	Heating Rate (K/min)	Ts (K)	Tf (K)	Tm (K)	(dα/dt)Max (%/Min)	Quality of Residue (%)
PE	10	728.05	748.05	757.45	30.05	0.30
	20	744.05	749.75	761.35	57.93	0.30
	30	748.85	753.15	765.15	82.74	0.30
PS	10	667.65	688.45	702.25	30.37	0.14
	20	680.65	696.75	716.75	54.84	0.14
	30	687.75	704.95	724.05	77.95	0.14
PS/PE	10	668.75	691.75	737.65	14.35	0.20
	20	683.05	703.75	751.75	28.64	0.20
	30	696.55	710.45	757.45	43.16	0.20

Note: Ts is the initial pyrolysis temperature, Tf is the termination pyrolysis temperature, Tm is the peak pyrolysis temperature, and (dα/dt) max is the maximum pyrolysis rate.

**Table 3:** Activation energy (E_α_), correlation coefficient (R_2_), and average activation energy (E_α_) of PE at different conversion rates calculated by the FWO equation, KAS equation, and Starink equation.

Conversion	FWO		KAS		Starink	
	E_α_	R_2_	E_α_	R_2_	E_α_	R_2_
0.1	234.7	0.9987	234.6	0.9986	234.9	0.9988
0.2	243.4	0.9991	244.2	0.9991	244.1	0.9993
0.3	255.1	0.9996	255.3	0.9994	256.2	0.9994
0.4	251.7	0.9999	251.3	0.9999	252.6	0.9999
0.5	253.4	0.9999	253.2	0.9999	253.5	0.9999
0.6	255.8	0.9999	256.5	0.9998	256.8	0.9996
0.7	250.2	0.9998	251.6	0.9996	251.5	0.9996
0.8	252.5	0.9994	252.9	0.9994	253.2	0.9994
0.9	249.0	0.9990	249.2	0.9989	249.5	0.9988
average	249.5	0.9995	249.9	0.9994	250.3	0.9994

**Table 4:** Activation energy (E_α_), correlation coefficient (R_2_), and average activation energy (E_α_) of PS at different conversion rates calculated by the FWO equation, KAS equation, and Starink equation.

Conversion	FWO		KAS		Starink	
	E_α_	R_2_	E_α_	R_2_	E_α_	R_2_
0.1	189.4	0.9989	188.9	0.9987	189.4	0.9986
0.2	192.2	0.9990	191.8	0.9994	191.6	0.9992
0.3	193.5	0.9993	193.1	0.9995	192.7	0.9995
0.4	196.4	0.9996	195.2	0.9996	196.2	0.9997
0.5	198.3	0.9999	198.1	0.9999	197.7	0.9999
0.6	200.5	0.9999	199.7	0.9999	200.3	0.9999
0.7	198.5	0.9997	198.2	0.9999	197.6	0.9999
0.8	199.2	0.9994	199.6	0.9996	198.4	0.9997
0.9	197.0	0.9994	197.6	0.9995	196.8	0.9993
average	196.1	0.9995	195.8	0.9996	195.6	0.9995

**Table 5:** Activation energy (E_α_), correlation coefficient (R_2_), and average activation energy (E_α_) of PS/PE at different conversion rates calculated by the FWO equation, KAS equation, and Starink equation.

Conversion	FWO		KAS		Starink	
	E_α_	R_2_	E_α_	R_2_	E_α_	R_2_
0.1	191.1	0.9996	190.6	0.999	191.6	0.9992
0.2	203.6	0.9996	203	0.9992	203.1	0.9994
0.3	207.1	0.9999	206.4	0.9996	206.8	0.9998
0.4	210.4	0.9999	209.3	0.9998	209.5	0.9999
0.5	214.9	0.9999	214.5	0.9999	214.6	0.9998
0.6	229.5	0.9999	229.0	0.9999	229.2	0.9998
0.7	224.3	0.9997	223.2	0.9999	223.8	0.9999
0.8	225.4	0.9998	224.8	0.9999	225.0	0.9997
0.9	221.6	0.9996	221.1	0.9998	221.3	0.9998
average	214.2	0.9998	213.5	0.9997	213.9	0.9997

**Table 6:** The pyrolysis product distribution and proportion of different experimental samples.

Samples	Pyrolysis Oil (g)	Percentage (%)	Pyrolysis Gas (g)	Percentage (%)	Residue (g)	Percentage (%)
PE	15.41	77.05	4.53	22.65	0.06	0.30
PS	17.33	86.65	2.63	13.15	0.04	0.20
Actual data						
PS/PE	17.96	89.80	2.00	10.00	0.04	0.20
Weighted data						
PS/PE	16.37	81.85	3.58	17.90	0.05	0.25

## Data Availability

Data is contained within the article.
